# Hungry bone syndrome and normalisation of renal phosphorus threshold after total parathyroidectomy for tertiary hyperparathyroidism in X-linked hypophosphataemia: a case report

**DOI:** 10.1186/1752-1947-8-84

**Published:** 2014-03-04

**Authors:** Rachel K Crowley, Mark Kilbane, Thomas FJ King, Michelle Morrin, Myra O’Keane, Malachi J McKenna

**Affiliations:** 1 Department of Endocrinology, St. Vincent’s University Hospital, Dublin, Ireland; 2 Metabolism Laboratory, St. Vincent’s University Hospital, Dublin, Ireland; 3 DXA Unit, St. Vincent’s University Hospital, Dublin, Ireland; 4 School of Medicine and Medical Sciences, University College Dublin, Dublin, Ireland

**Keywords:** Cinacalcet, Fibroblast growth factor 23, Hungry bone syndrome, Hyperparathyroidism, X-linked hypophosphataemia

## Abstract

**Introduction:**

This is the first report of which the authors are aware to describe this c.2166delinsGG mutation in X-linked hypophosphataemia and to describe normalisation of renal threshold for phosphate excretion after parathyroidectomy for tertiary hyperparathyroidism in X-linked hypophosphataemia.

**Case presentation:**

We present the case of a 34-year-old Caucasian woman with X-linked hypophosphataemia. She developed tertiary hyperparathyroidism with markedly high bone turnover requiring total parathyroidectomy and had prolonged requirement for intravenous calcium infusion after surgery. She had a novel mutation in her phosphate-regulating gene with homologies to endopeptidases on the X-chromosome and had an unusual degree of dependence on phosphate supplementation. Prior to operative intervention she had a trial of cinacalcet that improved bone turnover markers when used in isolation but which led to a paradoxical rise in parathyroid hormone levels when given with phosphate supplementation. After correction of hungry bone syndrome, the renal phosphorus threshold normalised as a manifestation of hypoparathyroid state despite marked elevation in level of fibroblast growth factor 23.

**Conclusions:**

This case illustrates the risk of tertiary hyperparathyroidism as a complication of treatment for hypophosphataemia; it highlights the morbidity associated with hungry bone syndrome and provides novel insight into renal handling of phosphorus.

## Introduction

X-linked hypophosphataemia (XLH) is a congenital cause of renal phosphorus wasting as a consequence of inactivating mutations in the phosphate-regulating gene with homologies to endopeptidases on the X chromosome (*PHEX*) that manifests with rickets and poor linear growth in childhood [1]. It has an X-linked dominant inheritance and the disease severity is variable; it is associated with increased bone expression of fibroblast growth factor 23 (FGF23) that mediates both the renal phosphorus wasting and the impaired activation of vitamin D to its hormonal form [1]. In childhood, it is treated by a combination of phosphorus supplements and activated forms of vitamin D, but judicious use of phosphorus is advised in order to prevent hyperparathyroidism [2].

We present the case of a woman with XLH who presented to adult services with evidence of tertiary hyperparathyroidism. We discuss the challenges in management of progressive severe hyperparathyroidism over a 16-year period and the unlikely benefit of the renal phosphorus handling by rendering her hypoparathyroid despite very high FGF23 levels.

## Case presentation

A 34-year-old Caucasian woman initially presented to the paediatric service at 19 months of age with difficulty walking and a broad-based gait, and was noted to be hypophosphataemic. She was commenced on vitamin D and phosphate replacement. On this treatment, she had normal growth and puberty, but had frontal bossing and enamel hyperplasia of her teeth. Her mature height was 158cm and her weight was 85.6kg. After transfer to the adult service, genetic testing identified a novel deletion-insertion mutation (c.2166delinsGG) in exon 22 of her *PHEX* gene; the mutation resulted in a premature termination at codon 725. Over the next 17 years, serial measurements were made of indices of calcium metabolism and bone turnover markers using previously described techniques [3].

**Figure 1:**
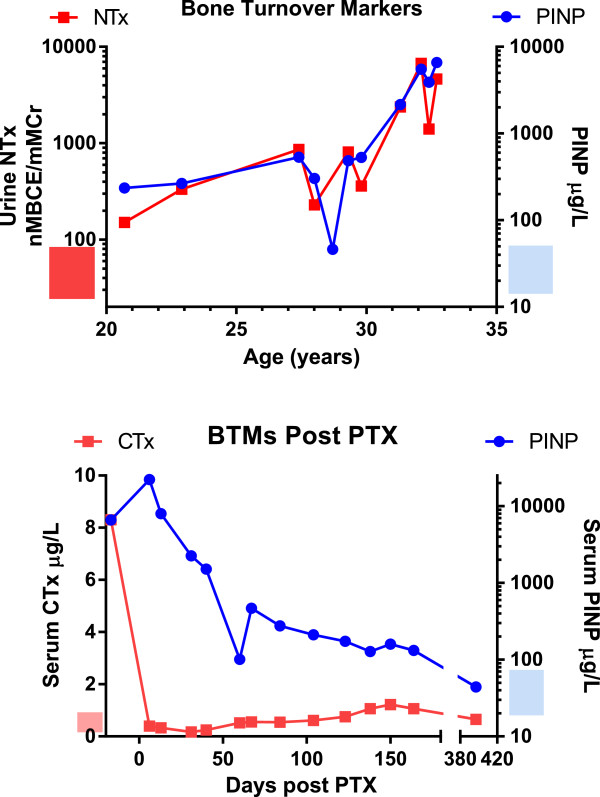
**Bone turnover markers pre- and post-parathyroidectomy. Top panel:** Serial changes prior to parathyroidectomy in bone resorption marker (urinary N-terminal cross-linking telopeptide of type I collagen) and a bone formation marker (serum procollagen type-I N-propeptide. **Lower panel:** Serial changes after parathyroidectomy in bone resorption markers (urinary C-terminal cross-linking telopeptides) and a bone formation marker (serum procollagen type-I N-propeptide). Reference ranges are denoted by stippled boxes along vertical axes. Abbreviations: BTM, bone turnover markers; CTxurinary C-terminal cross-linking telopeptides; mMCr, mmol creatinine; nMBCE, nmol Bone Collagen Equivalents; NTx, urinary N-terminal cross-linking telopeptide of type I collagen; PINP, serum procollagen type-I N-propeptide.

At the time of presentation to adult services she had evidence of tertiary hyperparathyroidism, with ionised calcium of 1.38mmol (reference range 1.19 to 1.35), and parathyroid hormone (PTH) of 454pg/mL (reference range 12 to 64). She was being treated with phosphorus supplementation in adulthood, of up to six tablets daily of Phosphate-Sandoz® 96mmol (or 3g of phosphorus); 1α-hydroxyvitamin D had been stopped due to hypercalcaemia. Attempts to reduce phosphorus supplementation failed because of muscle cramps and fatigue at lower doses. Over the next 7 years her clinical condition remained relatively stable, although monitoring of ionised calcium and PTH indicated that tertiary hyperparathyroidism continued to progress. Serial measurement of both a bone resorption marker, urinary N-terminal cross-linking telopeptide of type-I collagen, and a formation marker, procollagen type-I N-propeptide (PINP), were made throughout this time [3]; both levels rose steadily (Figure 1). When hypercalcaemia recurred (ionised calcium 1.57mmol/L, PTH 1107pg/mL), she agreed to stop phosphorus supplementation and to take a trial of cinacalcet therapy, starting at a dose of 30mg increasing to 60mg per day. Both ionised calcium (1.22mmol/L) and PTH (360pg/mL) dropped and bone turnover markers were reduced. However, she became wheelchair-bound secondary to myopathy that resolved on restarting phosphorus supplementation and ceasing cinacalcet. Cinacalcet was re-introduced with phosphorus after her clinical status improved, but this combination of therapies resulted in a paradoxical response (ionised calcium 1.21mmol/L but PTH 1084pg/mL) with concomitant surge in bone turnover markers to very high levels (Figure 1, age 27 to 28 years). In view of this PTH-mediated excess bone turnover, cinacalcet was discontinued. At this stage she complained of diffuse aches and pains.

Given the severity of parathyroid bone disease, persistent hypercalcaemia, and evidence of a slow deterioration in renal function with estimated glomerular filtration rate (eGFR) ranging from 59 to 80mL/minute/1.72m^2^ , (Modified Diet in Renal Disease method) it was decided to proceed with total parathyroidectomy. Since she was considered to be at high risk of severe hungry bone syndrome after parathyroidectomy, she was admitted prior to surgery for treatment with intravenous zoledronic acid that resulted in three separate adverse reactions: an acute phase response; a hypersensitivity response with mild angioedema followed by hepatic transaminitis, both of which responded to a short course of oral steroids; and symptomatic hypocalcaemia with total calcium 1.66mmol/L, necessitating intravenous calcium infusion. In view of the hypersensitivity response, this strategy of preparing the patient for total parathyroidectomy was abandoned. Admission for total parathyroidectomy was planned, with high-dependency admission for management of anticipated hungry bone syndrome.

She underwent open total parathyroidectomy. All four glands were enlarged, ranging in weight from 2.5 to 3.4g. Successful resection was confirmed by undetectable PTH levels. Persistent hypocalcaemia followed, necessitating intravenous calcium infusion as a hospital in-patient for 160 days post-surgery. A peripherally inserted central catheter line was placed and she was infused with 10% calcium gluconate solution to maintain total calcium over 1.9mmol/L. The total volume infused was 42,360mL, which was equivalent to 356g of elemental calcium. On discharge from hospital she was treated with 1α-vitamin D 6μg/day and oral calcium 2000mg daily; she no longer required phosphorus supplements.

**Figure 2:**
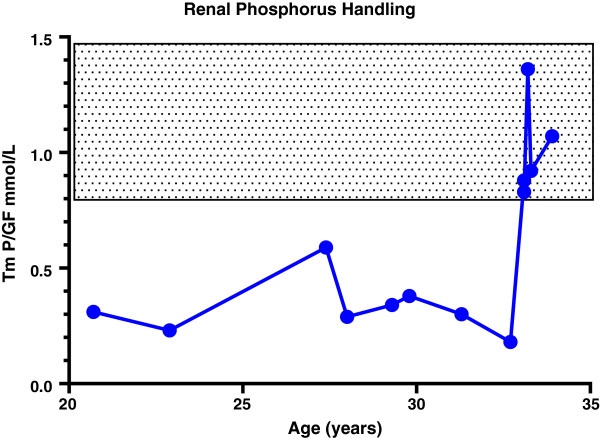
**Serial changes in renal phosphorus handling before and after parathyroidectomy.** Abbreviations: TmP/GF, theoretical renal phosphorus absorption threshold per glomerular filtrate.

Her bone turnover response was monitored in the postoperative period by measuring serum PINP and serum C-terminal cross-linking telopeptides (CTx). One year after surgery both PINP and CTx had returned to the normal reference range (Figure 1). Over the same time period she experienced a dramatic increase in bone mineral density (BMD): spine BMD increased by 68% up to Z-score of 7.2; and total hip BMD increased by 57% up to a Z-score of 5.2. Her gain in BMD and the reduction in CTX with increase in PINP reflects a prolonged spell of positive remodelling balance after total parathyroidectomy. Her theoretical renal phosphorus absorption threshold per glomerular filtrate (TmP/GF) increased from a low level to within the normal range (Figure 2). Her serum FGF23 was markedly elevated at 4790RU/mL (reference less than 100RU/mL); the level had not been measured preoperatively. Her postoperative course was complicated by deterioration in renal function that subsequently stabilised at an estimated GFR of 39mL/minute. There was no evidence of nephrocalcinosis.

## Discussion

The metabolic defect in XLH is an impairment of phosphorus reabsorption at the proximal tubule of the kidney and the underlying mutation is a loss of function of the *PHEX* gene [4]. Loss of function in *PHEX* is associated with increased circulating FGF23 which acts to reduce expression of sodium-phosphate co-transporters (NaPi) in the renal tubule in association with its co-factor Klotho, and to reduce 1α-hydroxylase activity [5,6]; the link between *PHEX* activity and FGF23 levels remains unclear. In childhood and adolescent cases with XLH, conventional treatment is with activated vitamin D and phosphorus [2]. Normalisation of serum phosphorus concentration is not an aim and is likely to result in secondary hyperparathyroidism [2].

High PTH leads to increased phosphorus excretion and thus increased requirement for supplementation; once this cycle has been initiated it is difficult to reverse. Tertiary hyperparathyroidism in XLH has been well described [7] and is thought to result from chronic stimulation of PTH by phosphorus supplements, leading to autonomous secretion from hyperplastic or adenomatous parathyroid glands [8]. In a previous case series by Savio *et al*. of patients with XLH who underwent parathyroidectomy for tertiary hyperparathyroidism, five of the six patients required either a one- or two-step total parathyroidectomy, with only one patient undergoing re-implantation of parathyroid tissue; and four demonstrated hungry bone syndrome postoperatively, although the most prolonged dependence on intravenous calcium was for 15 days [8]. The authors of the surgical case series also commented that postoperative hypoparathyroidism was a second insult to patients who already had mineral deficiencies from their inability to re-absorb phosphate; in our patient, TmP/GF normalised after parathyroidectomy. In Albright’s original description of XLH, one patient experienced transient normalisation of serum phosphorus after surgical excision of a hyperplastic parathyroid gland [9]. The normalisation of phosphate excretion in our patient may be due to the reduction in renal function; normalisation of TmP/GF has been reported in a patient with XLH with chronic kidney disease (CKD) previously [10]. However, a sudden dramatic rise in TmP/GF such as that of our patient (Figure 2) is not usually seen in CKD. The lack of requirement for phosphate supplementation in the hypoparathyroid state suggests that the presence of circulating PTH may be necessary for FGF23 to exert its phosphaturic action; this is in keeping with observations in the *Hyp* mouse with targeted deletion of the PTH gene [11]. Improvement in TmP/GF has been shown in patients with XLH receiving phosphorus who had a reduction in PTH on cinacalcet therapy [12].

Careful supplementation of phosphorus and vitamin D in childhood is necessary to optimise growth and to prevent development of tertiary hyperparathyroidism and renal impairment [2]. Phosphorus therapy is associated with diarrhoea and is a stimulant to PTH secretion [13] as well as FGF23 secretion [14]; it is associated with nephrocalcinosis in treated patients with XLH [15] and thus normalisation of serum phosphorus is not the target of therapy in XLH [2]. Vitamin D supplementation is associated with hypercalciuria [15], hence monitoring of urinary as well as serum calcium is necessary to assess whether vitamin D dosage should be reduced.

After development of tertiary hyperparathyroidism, therapeutic options become more limited. Clearly surgical management carries a high risk of hungry bone syndrome, and the benefits of total parathyroidectomy which might lead to an adynamic bone state must be weighed against the possibility of treatment failure if auto-transplant with a parathyroid fragment is attempted. Cinacalcet therapy may be of benefit; in other patient series phosphorus therapy was not discontinued and the paradoxical PTH rise experienced by our patient was not reported [16].

## Conclusions

This case report illustrates the challenges of management of XLH after tertiary hyperparathyroidism has developed, and supports improved liaison between paediatric and adult endocrine and rheumatology services to optimise care and prevent development of iatrogenic complications. The case also suggests a potential role for PTH in the phosphaturic action of FGF23, which will be of interest to endocrinologists and nephrologists.

## Consent

Written informed consent was obtained from the patient for publication of this case report and any accompanying images. A copy of the written consent is available for review by the Editor-in-Chief of this journal.

## Abbreviations

BMD: Bone mineral density; BTM: Bone turnover markers; CKD: Chronic kidney disease; CTx: C-terminal cross-linking telopeptide; FGF23: Fibroblast growth factor 23; eGFR: Estimated Glomerular filtration rate; mMCr: mmol creatinine; nMBCE: nmol Bone Collagen Equivalents; PHEX: Phosphate-regulating gene with homologies to endopeptidases on the X-chromosome; PINP: Procollagen type-I N-propeptide; PTH: Parathyroid hormone; TmP/GF: Theoretical renal phosphorus absorption threshold per glomerular filtrate; XLH: X-linked hypophosphataemia.

## Competing interests

The authors declare that they have no competing interests.

## Authors’ contributions

RC, TK and MMcK were responsible for collating clinical material and drafting the manuscript. MK, MM and MO’K were responsible for laboratory measurements. All authors read and approved the final manuscript.
